# Experimental and Analytical Studies on Low-Cost Glass-Fiber-Reinforced-Polymer-Composite-Strengthened Reinforced Concrete Beams: A Comparison with Carbon/Sisal Fiber-Reinforced Polymers

**DOI:** 10.3390/polym15194027

**Published:** 2023-10-09

**Authors:** Kittipoom Rodsin, Ali Ejaz, Qudeer Hussain, Rattapoohm Parichatprecha

**Affiliations:** 1 Center of Excellence in Structural Dynamics and Urban Management, Department of Civil and Environmental Engineering Technology, College of Industrial Technology, King Mongkut’s University of Technology North Bangkok, Bangkok 10800, Thailand; kittipoom.r@cit.kmutnb.ac.th; 2 National Institute of Transportation, National University of Sciences and Technology (NUST), Islamabad 44000, Pakistan; enggaliejax@gmail.com; 3 Dr. House Consultants Co., Ltd., Bangkok 10330, Thailand; ebbadat@hotmail.com; 4 Department of Civil Engineering, School of Engineering, King Mongkut’s Institute of Technology Ladkrabang, Bangkok 10520, Thailand

**Keywords:** shear, strengthening, GFRP, configurations, u-shaped, side-bonded, models

## Abstract

This study presents an experimental framework with seventeen beams to investigate the impact of loading type, configuration, and through-bolt anchorage on LC-GFRP (Low-Cost Glass-Fiber-Reinforced Polymer) confinement performance. Beams underwent three-point and four-point bending, with LC-GFRP applied in various ways, including U-shaped, side-bonded, and fully wrapped, with and without anchors. The performance of LC-GFRP was compared to CFRP (Carbon-Fiber-Reinforced Polymer) and sisal wraps. LC-GFRP in side-bonded and U-shaped configurations without anchors under three-point bending showed no shear failure, while those under four-point bending without anchors experienced shear failure. With anchors, U-shaped configurations successfully prevented shear failure. The side-bonded, U-shaped, and U-shaped configurations along the full span with anchors demonstrated peak capacity enhancements of 72.11%, 43.66%, and 68.39% higher improvements than the corresponding configurations without anchors, respectively. Wrapping all sides of the beam with LC-GFRP or CFRP prevented shear failure without additional anchors, with complete wrapping being the most efficient method. When anchors were used, significant capacity enhancements were observed. Existing shear strength prediction models were evaluated, highlighting the need for more tailored expressions for LC-GFRP confinement, especially for non-U-shaped configurations.

## 1. Introduction

The need to meet the higher strength requirements of revised design codes and the degradation of structures over time due to environmental effects are among the various factors driving the reinforcement of existing infrastructures around the world. Old concrete structures often fail to comply with modern seismic design codes, showing inadequate shear capacity and ductility. This was evident in the Hyogoken-Nanbu Earthquake of 1995, where concrete structures, especially reinforced concrete piers and rigid frames in elevated highways, suffered considerable damage. Upgrading these structures is essential to improve their resilience against future natural disasters [1,2].

Reinforced concrete (RC) elements that experience shear stresses are vulnerable to brittle failures. The resistance of these elements depends mainly on the transfer of shear stresses through a rough crack, which is commonly termed as “aggregate interlock” [3,4]. The complex nature of the shear phenomenon makes it difficult to be predicted accurately. Therefore, empirical or semi-empirical expressions are utilized by many practical codes to determine the shear capacity of RC beams [5]. The shear capacity of an RC beam comprises contributions from concrete and internal transverse reinforcements to transform the brittle behavior to ductile. Recently, Fiber-Reinforced Polymers (FRPs) have gained significance over conventional steel reinforcement, ascribed to them being lightweight, resistant to corrosion, and easy to apply, as well as having more dependable bond line strength than steel [6]. 

The debonding of FRP from the concrete surface has been a typical failure [7]. Since the 1990s, numerous studies have employed FRPs for the strengthening of RC members [8,9,10,11]. The findings suggest that when beams are strengthened, the primary modes of shear failure are either through the tensile rupture of the FRP or due to the debonding of the FRP from the sides of the RC beam. The specific mode of failure is affected by the method employed in the beam-strengthening process [12]. The bond strength between FRP and concrete governs the maximum stress developed in FRP, and this bond strength is determined to be significantly lower than the strength of FRP [13]. In addition, it is worth noting that FRP debonding failure tends to be brittle, resulting in catastrophic failure with relatively low structural deformation [14,15]. Consequently, current design guidelines for FRP strengthening recommend the use of additional anchorage systems in combination with FRP wraps to inhibit their debonding [16]. 

The issue with the debonding of FRP within shear dominant regions has been highlighted in previous studies. Several researchers have documented attempts to enhance the shear capacity of shallow [17,18,19,20,21] and deep beams [22,23] via the application of externally bonded FRPs. Triantifillou [24] performed shear strengthening of eleven RC beams using epoxy-bonded FRP composites. The performance of strengthened beams was superior to the reference specimens. Nevertheless, FRP debonding was observed in all beams, reducing the efficiency of FRP composites. Monti et al. [25] performed an experimental framework on twenty-four RC beams with shear-deficient characteristics. The shear strengthening was performed by employing FRP composites in various configurations. Interestingly, the debonding of FRP composites was observed in all strengthened beams, irrespective of the type of FRP configuration. Baggio et al. [26] employed FRP systems composed of carbon, glass, and a fiber-reinforced cementitious matrix. A total of nine shear-deficient RC slender beams were tested. It was observed that the presence of anchors in combination with FRP wraps significantly enhanced the ductility, and the debonding of FRP wraps was prevented. In Boyd’s study [27], the influence of different types of sprayed FRP (SFRP) strengthening schemes on the shear strength of shallow RC beams was investigated. Three schemes were considered: A, C, and D. Scheme A involved SFRP applied only to the two sides of the beam, scheme C comprised SFRP applied to the sides and bottom faces, and scheme D encompassed SFRP applied to all faces of the beam. The results demonstrated that schemes C and D were more effective in enhancing the shear strength compared to scheme A. Regarding failure modes, beams strengthened with schemes A and C failed due to the debonding of the SFRP, while specimens with scheme D experienced failure through the rupture of the SFRP. Arslan et al. [28] utilized anchored and non-anchored CFRP fabrics for the shear strengthening of T-beams and observed up to a 54% increase in shear capacity using a combination of anchors and CFRP strips. Aksoylu et al. [29] enhanced the shear capacity of beams with circular openings by employing CFRP composites and found CFRP composites to be ineffective when the ratio of the hole diameter to beam height reached 0.64. 

Soleimani and Banthia [30] investigated the performance of a SFRP for the shear strengthening of RC beams. To enhance the bond between the concrete surface and the SFRP, they employed an anchoring technique using bolts and nuts along with a roughened concrete surface. The proposed anchorage technique was relatively cheaper and easier to install than conventional FRP anchorages. The research findings determined that a roughened concrete surface, in conjunction with a bolt anchoring system, proved to be effective in increasing the bond between the concrete and the SFRP. Hussain and Pimanmas [31] extended the application of the through-bolt anchorage system to enhance the shear performance of RC deep beams. The type of SFRP, its thickness and configuration, and the compressive strength of concrete were the parameters of interest. The findings revealed that a strengthened beam without additional anchorage demonstrated similar behavior as that of the unstrengthened beam, whereas the provision of through-bolt anchorages substantially enhanced the shear capacity. In another study, Hussain and Pimanmas [32] conducted experiments on twenty-nine RC deep beams with openings. The combination of SFRP with mechanical anchorages was found to be effective in enhancing the shear capacity. 

The findings mentioned above suggest that the combination of through-bolt anchors and external wraps could effectively enhance the shear capacity by preventing premature debonding of the wraps. However, the use of through-bolt anchors has been limited to deep beams thus far. This study aims to evaluate the performance of through-bolt anchors in preventing the debonding of carbon FRP (CFRP) or low-cost glass FRP (LC-GFRP) wraps when applied to resist brittle shear failure in shallow beams. LC-GFRP offers several advantages, such as its relatively low cost, easy availability, and high tensile strength. Yoddumrong et al. [33] demonstrated the use of LC-GFRP in strengthening RC columns, while Rodsin et al. [34] utilized LC-GFRP to enhance the compressive strength of extremely low-strength concrete. Later, Rodsin et al. [35] observed significant improvements in the compressive behavior of recycled aggregate concrete through the use of LC-GFRP. Joyklad et al. [36] also noticed significant improvements in the compressive behavior of recycled brick aggregate concrete via the application of external LC-GFRP sheets. These studies have shown that LC-GFRP can serve as an effective and viable alternative to CFRP in strengthening applications. However, its potential in shear-strengthening applications has not been explored. Another important property of LC-GFRP is that it offers a bidirectional layout of fibers that has proven to be more effective than unidirectional fibers in improving the shear resistance of RC members [37,38]. Therefore, this study aims to employ LC-GFRP for the shear strengthening of shallow beams. For comparison, shear strengthening with CFRP and natural sisal FRP jackets will also be conducted. Sisal fiber is one of the most widely used natural fibers and is very easily cultivated. Sisal fiber is a hard fiber extracted from the leaves of the sisal plant (Agave sisalana). In Thailand, the recent market price of the LC-GFRP is USD 1.0, which is 60.0% and 350.0% less than the conventional natural sisal FRP and CFRP, respectively [34]. Furthermore, different configurations of CFRP and LC-GFRP will be utilized under three-point or four-point bending to assess their performance. At present, LC-GFRP has only been utilized for strengthening the axial compressive properties of concrete. The authors acknowledge that the efficacy of LC-GFRP needs to be explored for structural applications, especially for RC members subjected to flexure-dominated or shear-dominated forces. This will help in identifying the true potential of LC-GFRP and its applicability for structural strengthening.

## 2. Experimental Program

### 2.1. Material Properties

**Figure 1:**
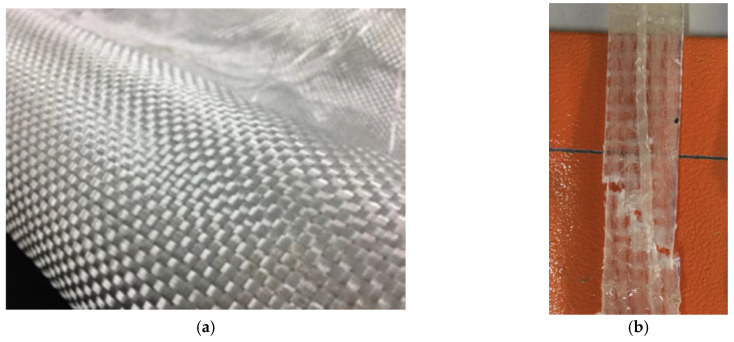
(**a**) Bidirectional LC-GFRP and (**b**) failure patterns of LC-GFRP.

Ready-mix concrete was used to cast all beams. The target compressive strength was 30 MPa. Ordinary Portland cement was used, whereas the maximum size of coarse aggregates was 19 mm. LC-GFRP sheets were developed using locally available bidirectional glass fibers (600 g/m^2^), as shown in Figure 1a. Standard tensile coupons were prepared from LC-GFRP sheets to evaluate their mechanical properties. The failure mode of LC-GFRP sheets is depicted in Figure 1b. Five coupons of LC-GFRP and sisal FRP were tested, following the guidelines of ASTM D3013-13 [39]. The length and width of tensile coupons were 200 mm and 20 mm, respectively. A computer-controlled universal testing machine M500-50AT manufactured by Testometric, Rochdale, England, was used to perform tensile tests. The tests were performed using the displacement control method. The mechanical properties of LC-GFRP sheets, sisal FRP, and epoxy resin are summarized in Table 1. The tensile coupons were prepared using a single layer of each fiber. The thicknesses of the tensile coupons were approximately 2–3 mm along with epoxy adhesion. The properties of CFRP were provided by the manufacturer, as listed in Table 1. Carbon fibers have very high tensile strength as compared to the fibers in LC-GFRP, resulting in a significantly greater elastic modulus than that of LC-GFRP.

The top longitudinal reinforcement comprised two 12 mm diameter deformed rebars, whereas three 12 mm diameter deformed rebars were used for the bottom longitudinal reinforcement. The transverse reinforcement comprised 6 mm plain rebars placed at 90 mm and 140 mm at the center in Group A and Group B, respectively. In groups A and B, the longitudinal reinforced ratio was 0.0313 and the balanced reinforcement ratio was 0.0283. Further, in group B, the stirrup volumetric ratio was 0.0395.

### 2.2. Details of Test Specimens

**Figure 2:**
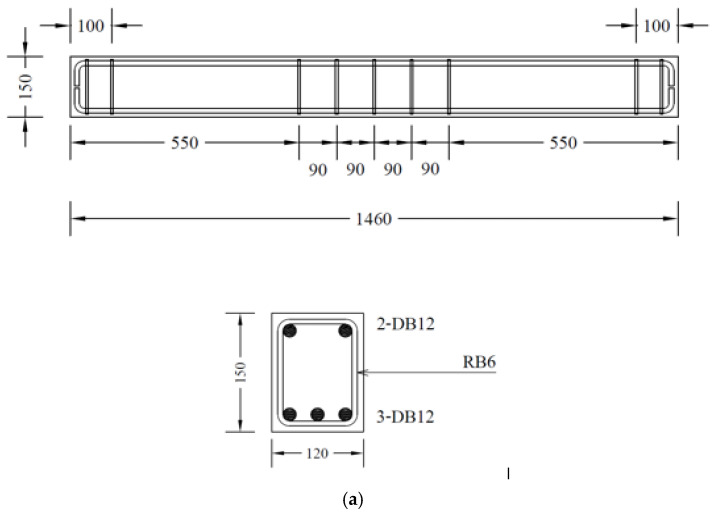

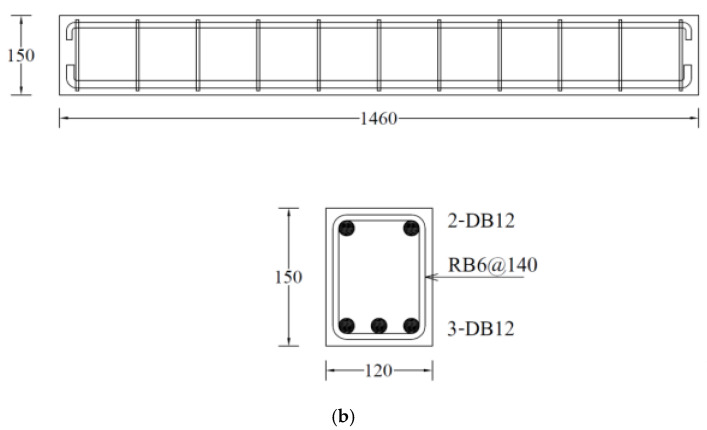
Structural details of tested beams in (**a**) Group A and (**b**) Group B.

This study comprised an experimental program on seventeen RC beams, categorized mainly into two groups, as presented in Table 2. The details of beams are shown in Figure 2. Larger beam specimens representing actual beam dimensions in structural frames could provide more realistic and reliable results; however, in this study, the sizes of beam specimens were adopted following the ultimate load capacity of the load cell, hydraulic jack, and reaction frame. The two groups were formed on the basis of the configuration of stirrups, as shown in Figure 2. Beams in Group A comprised stirrups within the middle zone, i.e., no stirrups were provided within shear spans. In comparison, beams in Group B incorporated stirrups within shear spans. Moreover, seven beams were tested in Group A. Three beams were tested under three-point bending, whereas the remaining four beams were tested under four-point bending. The details of beams tested in Group A are as follows: for beams tested under three-point bending, one beam was tested in as-built conditions, whereas the remaining two beams were strengthened with two layers of LC-GFRP. However, the configuration of LC-GFRP differed in two beams, i.e., one beam was strengthened with LC-GFRP bonded to the sides only (SB), whereas a U-shaped configuration was adopted in the other beam. For beams tested under four-point bending, one was tested in as-built conditions, whereas SB, U-shaped (U), and U-shaped along the whole span (UF) configurations were adopted for the remaining three beams. It must be noted that LC-GFRP was used for the strengthening of beams in Group A. 

**Figure 3:**
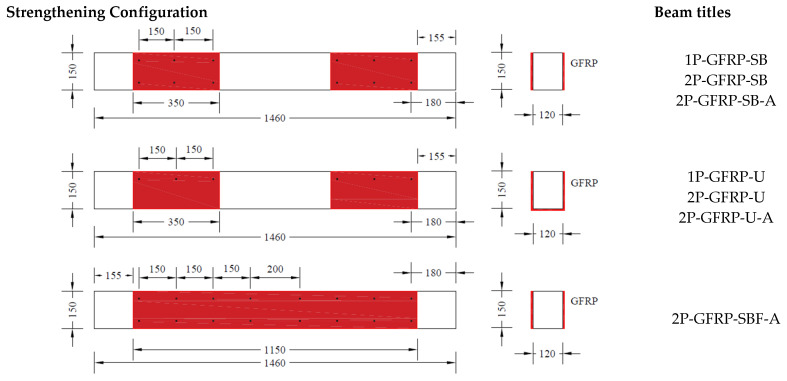

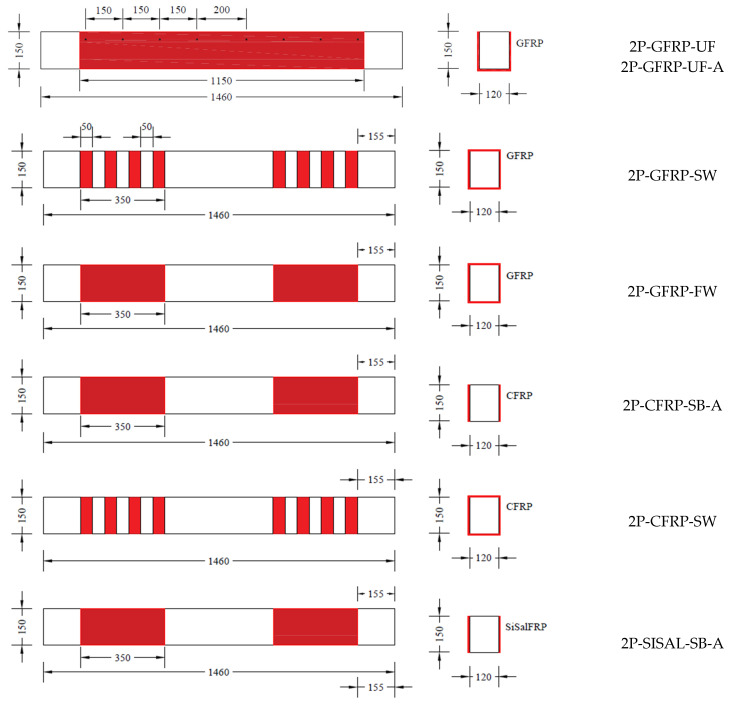
Different configurations adopted for strengthening.

Group B comprised ten beams tested under four-point bending. One beam was tested in as-built conditions. Six beams were strengthened with LC-GFRP using different configurations, including SB, U, UF, side-bonded along the whole span (SBF), strips within the shear span (SW), and fully wrapped (FW). Two beams were strengthened with CFRP in SB and SW configurations, whereas one beam was strengthened with sisal FRP in the SB configuration. Sisal fiber production involves harvesting mature sisal leaves, decorticating them to extract fibers, grading and sorting the fibers, and then baling and packaging them for distribution [40]. It is noteworthy that the strengthening was performed using two layers for LC-GFRP and sisal jackets, whereas a single layer was used in the case of CFRP. Further, no anchors were used for SW and FW configurations. In addition, strengthening with CFRP and sisal jackets did not accompany anchors. The locations of anchors are depicted in Figure 3 for each configuration type.

### 2.3. Instrumentation and Test Setup

**Figure 4:**
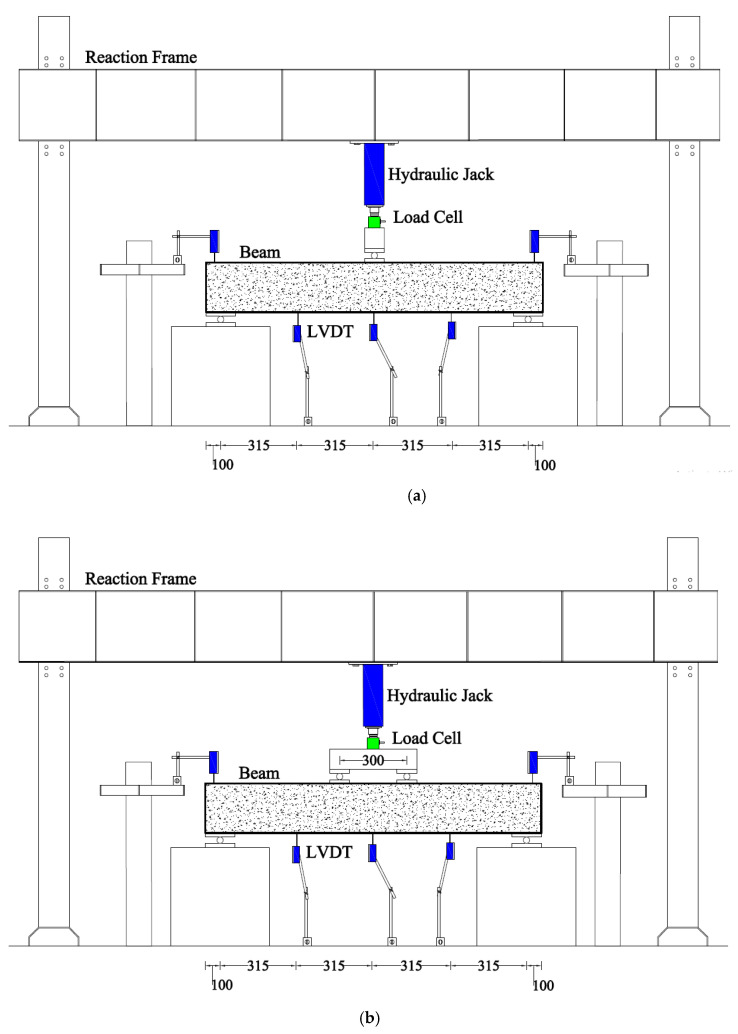
Typical test setup for (**a**) three-point and (**b**) four-point bending.

Three-point and four-point loads were applied to specimens in Group A, whereas specimens in Group B experienced four-point bending only. The load was applied in a reaction that had a capacity of 2000 kN. The load was applied using a hydraulic jack with a capacity of 500 kN. The vertical deflection was monitored using a linear variable differential transducer placed concentrically under the beams. A typical test setup is shown in Figure 4. One strain gauge was attached to the bottom longitudinal bar at the centerline of each beam. The beam installation was performed using an overhead crane and it took approximately 1 h to install the beam on the supports. The LVDT settlements were recorded using a data logger and a computer. In this study, the middle data of middle LVDT were used to plot load versus deflection graphs because of maximum deflection at this LVDT. The shear span to depth ratios were 4.2 and 3.2 for beams in Group A and B, respectively. 

### 2.4. Strengthening Process

**Figure 5:**
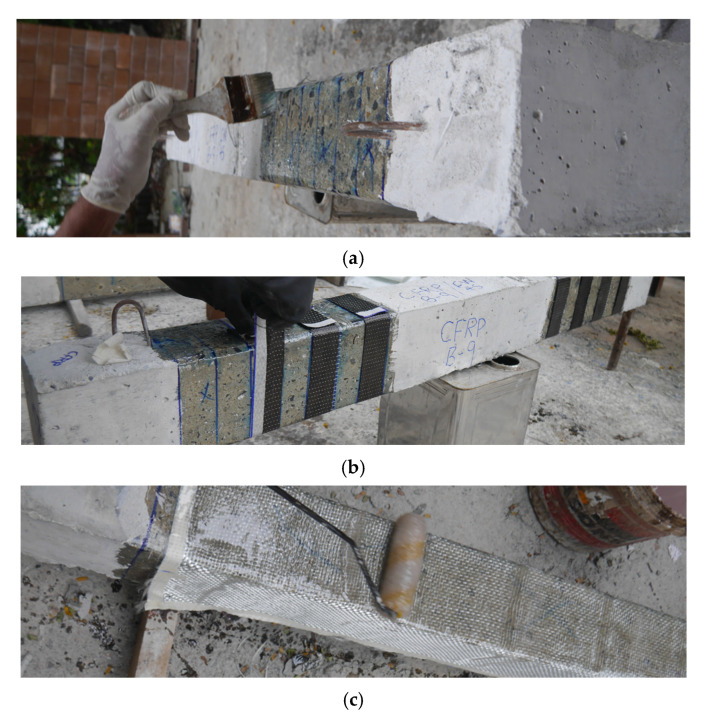
Strengthening process: (**a**) soaking the concrete surface with epoxy, (**b**) application of epoxy-impregnated CFRP strips, and (**c**) impregnating the surface of the existing layer before the application of the second layer.

External wraps were applied using a wet layout process, as shown in Figure 5. The surface of the beams was roughened uniformly using a hammer and chisel to enhance the bond between external wraps and the concrete surface. Also, the sharp corners of beams were rounded (corner radius 13 mm) to avoid stress accumulation at the shared corners. Then, the epoxy resin was applied using a hand brush. The epoxy resin was formed of two parts, i.e., resin and hardener. Resin and hardener were mixed using a ratio of 2:1 (resin/hardener). Then, fully epoxy-impregnated fiber sheets were applied at the marked locations. For the application of subsequent layers, the surface of already-applied layers was soaked with epoxy using a hand roller, followed by the application of second layers. During the epoxy application, proper care was taken to ensure that the fibers were 100% fully saturated with the epoxy resin.

**Figure 6:**
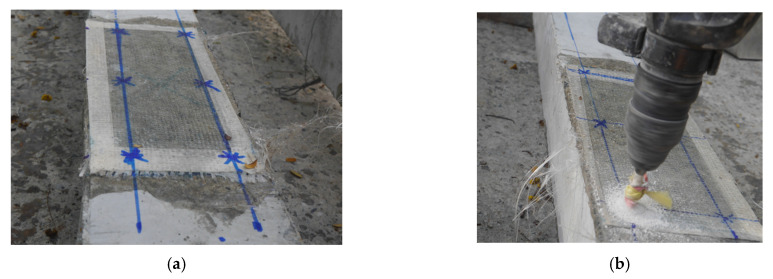

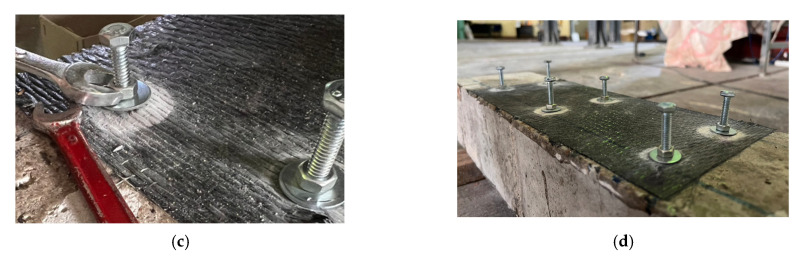
Installation of anchors (**a**) marking the exact locations, (**b**) drilling holes, (**c**) tightening nuts, and (**d**) through-bolt anchors installed.

The application of anchors was performed via the following steps: the location of anchors was clearly marked on external wraps (see Figure 6a), holes were drilled at the marked locations (see Figure 6b), anchor holes were cleaned using air compressor pressure, threaded rods of 8 mm diameter were inserted within holes, and washers and nuts were placed and manually tightened (see Figure 6c). See the properties of the anchorage system below.

## 3. Experimental Results

### 3.1. Failure Modes

**Figure 7:**
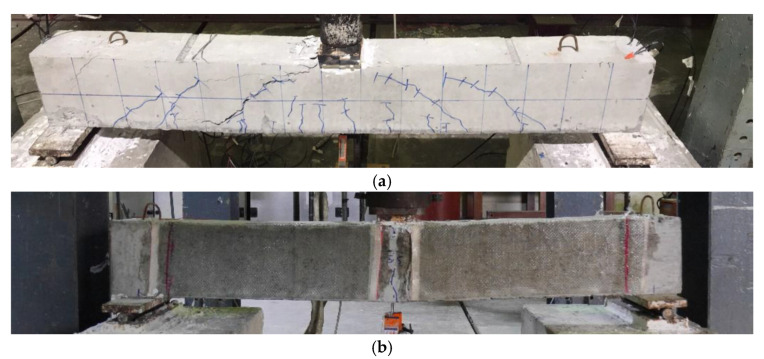
Failure modes of beams in Group A subjected to three-point bending: (**a**) 1P-CON, (**b**) typical of 1P-GFRP-SB and 1P-GFRP-U.

The failure modes of beams in Group A subjected to three-point bending are shown in Figure 7. It must be noted that no anchors were used to comprehend LC-GFRP confinement in Group A beams. The failure of Beam 1P-CON was shear failure attributed to the sudden formation of diagonal cracks, leading to the drop in load-carrying capacity, as shown in Figure 7a. On the contrary, the failure of beams 1P-GFRP-SB and 1P-GFRP-U exhibited flexural failure without demonstrating debonding of the GFRP layers, as shown in Figure 7b.

**Figure 8:**
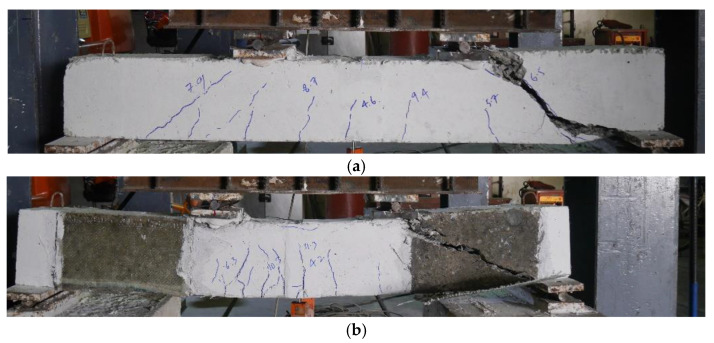
Failure modes of beams in Group A subjected to four-point bending: (**a**) 2P-CON-01, (**b**) typical of 2P-GFRP-SB, 2P-GFRP-U, and 2P-GFRP-UF.

The failure modes of beams in Group A subjected to four-point bending are shown in Figure 8. The control beam, as expected, demonstrated brittle shear failure characterized by large diagonal cracks originating from the bending point to the supports (see Figure 8a). The failure of beams 2P-GFRP-SB, 2P-GFRP-U, and 2P-GFRP-UF was characterized by the debonding of LC-GFRP layers and large shear cracks. It is important to observe that SB and U-shaped configurations (without anchors) did not debond under three-point bending in addition to altering the shear failure to flexural failure. In comparison, SB and U-shaped configurations (without anchors) could not prevent debonding and shear failure under four-point bending. This can be attributed to the smaller shear spans under the four-point bending as compared to three-point bending, leading to higher shear demand in the case of four-point bending (Figure 8b). 

**Figure 9:**
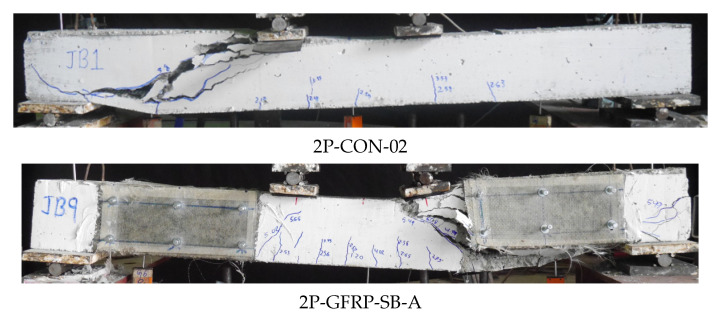

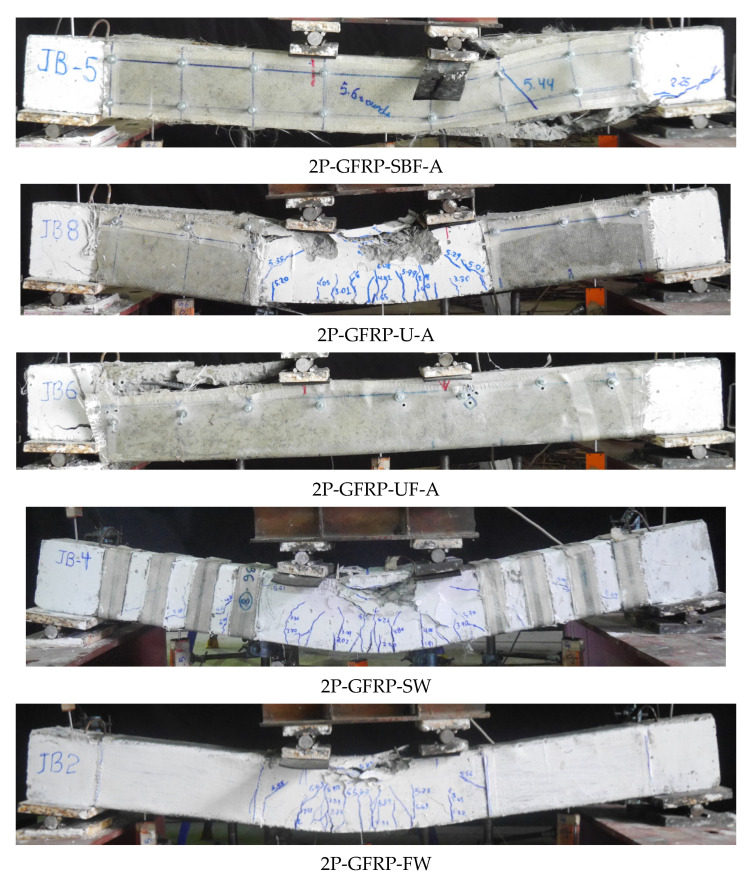
Failure modes of LC-GFRP-strengthened beams in Group B.

The failure modes of beams in Group B with LC-GFRP strengthening are shown in Figure 9. The control Beam 2P-CON-02 exhibited brittle shear failure with noticeable shear cracks. The failure of beams 2P-GFRP-SB-A and 2P-GFRP-SBF-A was accompanied by debonding of LC-GFRP on one end, and noticeable shear cracks were observed, suggesting that only a partial improvement in the shear behavior was obtained. The failure of Beam 2P-GFRP-U-A did not exhibit debonding or shear cracks. This indicates that two layers of GFRP applied in a U-shaped configuration and supplemented with through-bolt anchors effectively prevented brittle shear failure. It is noteworthy that Beam 2P-GFRP-U (i.e., strengthened with U-shaped LC-GFRP without anchors) had failed in shear strength, and debonding was observed. This emphasizes the importance and efficacy of through-bolt anchors in preventing the debonding of LC-GFRP layers that prevented the premature capacity loss of Beam 2P-GFRP-U-A. The performance of beams 2P-GFRP-SW and 2P-GFRP-FW was also flexure-dominated and did not exhibit debonding or shear cracks. Unlike Beam 2P-GFRP-U-A, the failure of 2P-GFRP-UF-A was triggered by debonding and ultimately failed in shear strength. This can be attributed to the enhanced flexural capacity within the constant moment region in Beam 2P-GFRP-UF-A due to the presence of LC-GFRP layers and anchors.

**Figure 10:**
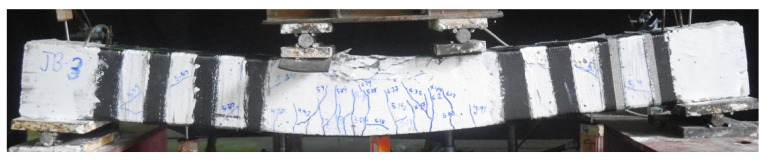
Failure modes of CFRP-strengthened beams.

**Figure 11:**
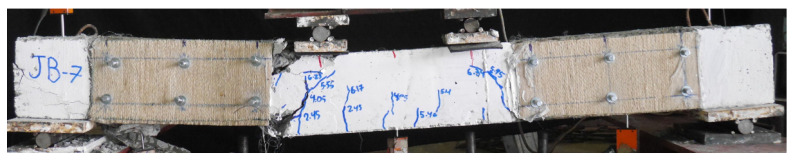
Failure mode of sisal-strengthened beam 2P-SISAL-SB-A.

The failure of the CFRP-strengthened beams is shown in Figure 10. It must be noted that Beam 2P-CFRP-SW was strengthened with strips of CFRP on four sides. The corresponding flexure-dominated behavior that was exhibited was characterized by numerous vertical cracks within the constant moment region, as shown in Figure 10. A few minor shear cracks were observed, whereas concrete crushing was observed within the constant moment region. On the contrary, the behavior of Beam 2P-CFRP-SB-A was shear-dominated, as characterized by the formation of large shear cracks. It is interesting to observe that a single wrap of CFRP could not prevent shear failure in a side-bonded configuration. The performance of the sisal-strengthened beam was subpar and demonstrated shear failure without the debonding of the sisal FRP, as shown in Figure 11.

### 3.2. Load versus Deflection Response

**Figure 12:**
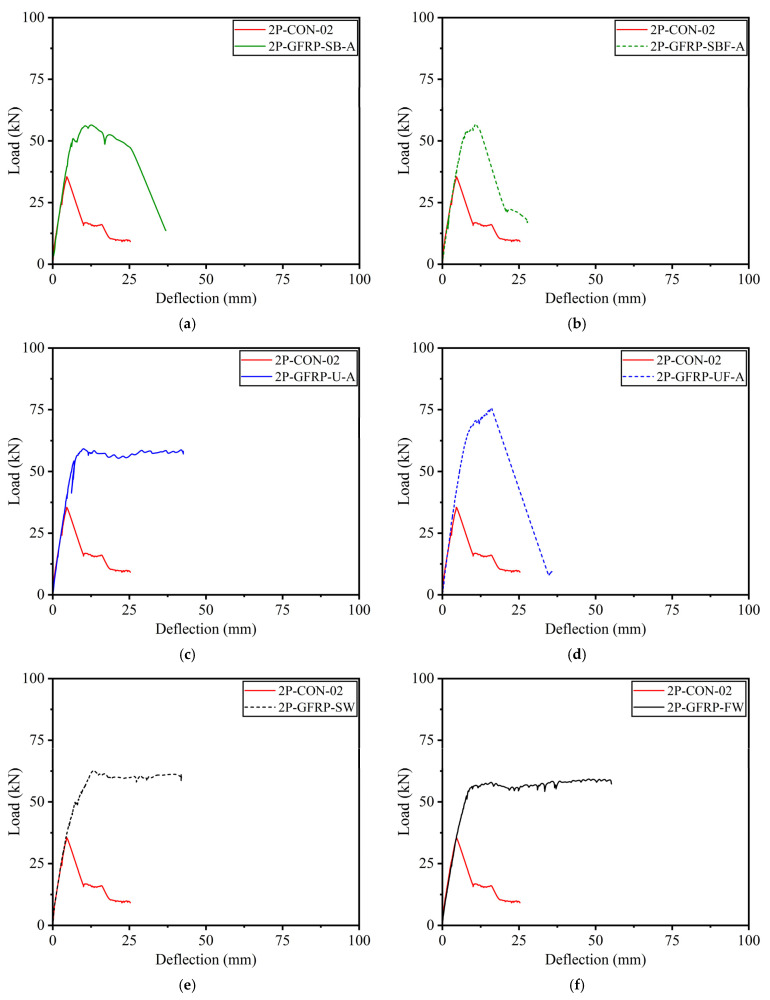
Comparison of LC-GFRP-strengthened Group B beams with control beams: (**a**) 2P-GFRP-SB-A, (**b**) 2P-GFRP-SBF-A, (**c**) 2P-GFRP-U-A, (**d**) 2P-GFRP-UF-A, (**e**) 2P-GFRP-SW, and (**f**) 2P-GFRP-FW.

**Figure 13:**
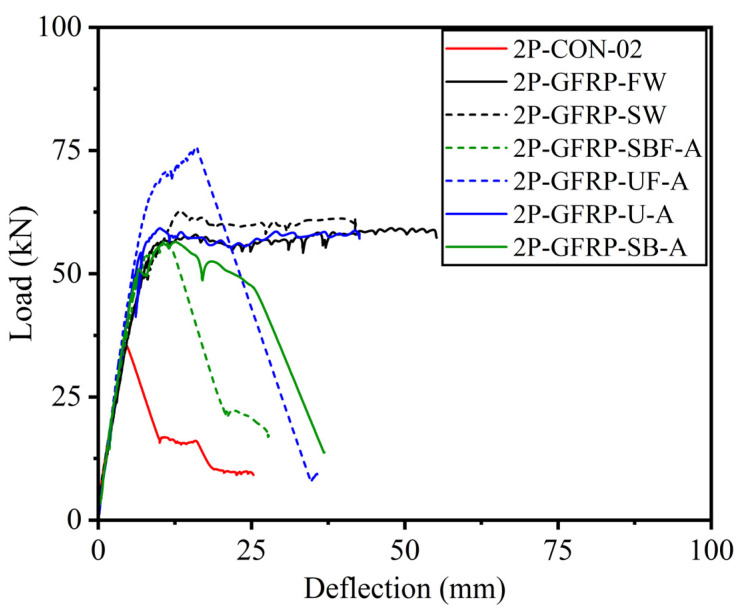
Comparison of load–deflection response of LC-GFRP-strengthened Group B beams.

The load versus deflection curves for GFRP-strengthened Group B beams are shown in Figure 12, in comparison to the corresponding control Beam 2P-CON-02. It can be seen that beams 2P-GFRP-SB-A (side-bonded configuration within shear span) and 2P-GFRP-SBF-A (side-bonded configuration along full span) demonstrated higher peak loads than the control beam. In comparison, the ductility of Beam 2P-GFRP-SBF-A was lower than Beam 2P-GFRP-SB-A. Nonetheless, both beams suffered shear failure, as can be seen in their abrupt drop in capacities. This implies that the application of side-bonded LC-GFRP was insufficient in altering the brittle failure to be a ductile failure. Next, the load–deflection curves of beams 2P-GFRP-U-A (U-shape configuration within shear span) and 2P-GFRP-UF-A (U-shape configuration along full span) are compared in Figure 12c,d, respectively. The failure mode of Beam 2P-GFRP-U-A exhibited neither debonding nor shear failure. This is reflected in the corresponding load–deflection curve in Figure 12c, where an abrupt drop in capacity was not observed. On the contrary, a U-shaped configuration along the full span could not prevent shear failure (see Figure 12d). Finally, the load–deflection curves of beams 2P-GFRP-SW and 2P-GFRP-FW are shown in Figure 12e,f, respectively. Both of these beams were wrapped on their four sides. However, the behavior of a fully wrapped beam (i.e., 2P-GFRP-FW) was not superior to that of the strip-wrapped beam (i.e., 2P-GFRP-SW). Finally, a comparison of load–deflection curves of all LC-GFRP-strengthened beams is shown in Figure 13. The following observations could be made: the performance of the side-bonded configuration, either along the full span or within the shear span only, was inferior to the U-shaped configuration, especially in terms of the ductility achieved. A U-shaped configuration along the full span imparted a significant increase in the peak load, but it increased the shear demand as well, resulting in an abrupt drop in load capacity. It may be noted that the provision of either U-shaped or side-bonded configurations outside the shear stress had adverse effects on the performance of LC-GFRP confinement.

**Figure 14:**
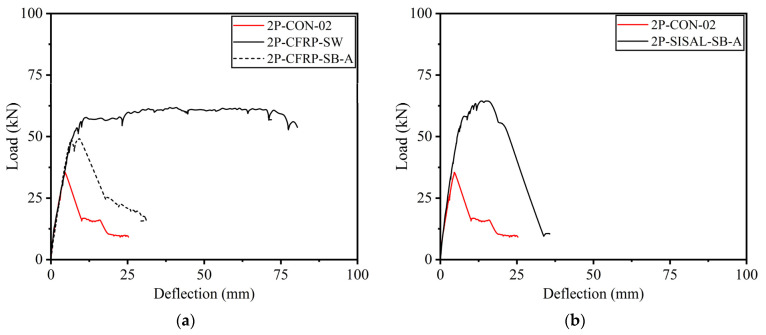
Load–deflection curves of Group B beams: (**a**) CFRP-strengthened and (**b**) sisal-strengthened.

The load–deflection curves of CFRP-strengthened beams in Group B are shown in Figure 14a. The side-bonded configuration did not impart a significant improvement, mainly in terms of ductility, whereas a noticeable improvement in peak load capacity was observed. On the contrary, CFRP strips (applied on all four sides) imparted significant improvements in ductility as well as peak load capacity. The application of sisal in a side-bonded configuration also resulted in subpar structural behavior, as shown in Figure 14. It is noteworthy that the maximum deflection encountered in sufficiently confined beams, i.e., 2P-GFRP-SW, 2P-GFRP-FW, 2P-GFRP-U-A, and 2P-CFRP-SW, was limited by the stroke limit of the used LVDTs. Nonetheless, the large deflection without a drop in load-carrying capacity suggests the usefulness of fully wrapped and U-shaped configurations in altering the brittle shear failure. In particular, the application of U-shaped confinement supplemented with through-bolt anchors is useful, particularly considering the practical aspects of RC beams, as it becomes difficult to wrap beams in existing structures from all four sides due to the presence of slabs.

**Figure 15:**
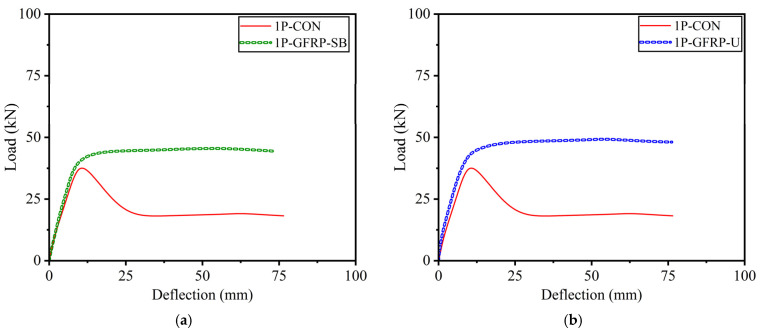
Load–deflection curves of Group A beams subjected to three-point bending: (**a**) side-bonded configuration and (**b**) U-shaped configuration.

The load–deflection curves of beams in Group A subjected to three-point bending are shown in Figure 15. It is vital to recall that no anchors were used to supplement LC-GFRP sheets in Group A beams. Despite the absence of anchors, stable load–deflection curves were observed until there were large deflections. However, this is mainly ascribed to the nature of loading, i.e., three-point bending, which constitutes large shear spans, resulting in lower shear demands. The consequence is reflected in Figure 15, demonstrating that as the length of the shear span is increased, the demand for through-bolt anchors to prevent debonding and shear failures is reduced.

**Figure 16:**
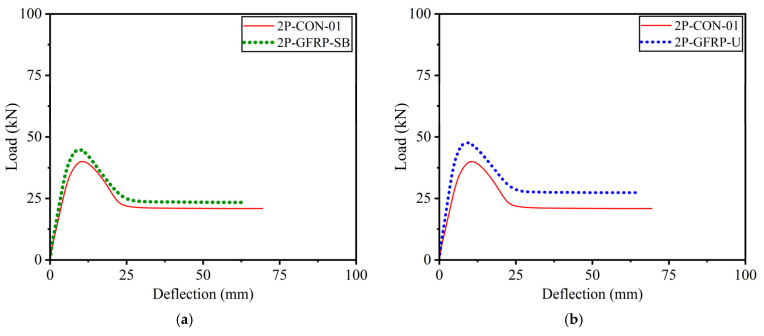

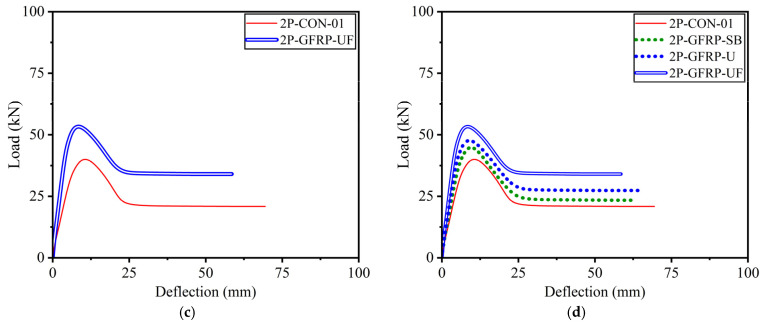
Load–deflection curves of Group A beams subjected to four-point bending: (**a**) side-bonded configuration, (**b**) U-shaped configuration applied to shear span only, (**c**) U-shaped configuration applied to full span, (**d**) comparison of all beams.

The load–deflection curves of beams in Group A that experienced four-point bending are shown in Figure 16. The post-peak behavior of the curves demonstrated a drop in capacity that can be attributed to the debonding of LC-GFRP, as presented in their corresponding failure modes. Nonetheless, the improvement in ductility was negligible, highlighting the importance of through-bolt anchorage to supplement LC-GFRP confinement.

### 3.3. Improvement in Peak Loads

**Figure 17:**
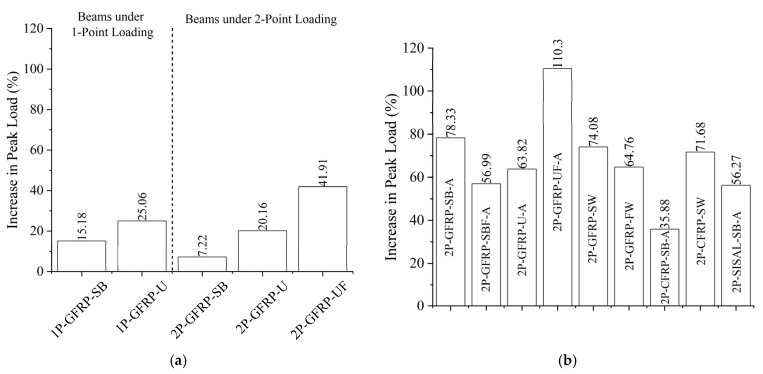
Increase in peak load: (**a**) Group A beams and (**b**) Group B beams.

A summary of experimental results is presented in Table 3. The shear capacity calculated using ACI 318-19 [3] recommendations was 37 kN and 33 kN for Group A and Group B beams, respectively. This corresponds to the measured compressive strength of 38 MPa and 32 MPa, respectively. The shear-dominated behavior often undermines the load-carrying capacities of RC members. The strengthening of shear-critical members involves two objectives: to improve the load-carrying capacities and to improve the structural behavior by altering the brittle failure to become a ductile failure. The improvement in the load-carrying capacities due to the application of LC-GFRP, CFRP, or sisal wraps on Group A beams is depicted in Figure 17a. It can be seen that Group A beams subjected to three-point bending exhibited a slight improvement, whereas the same beams (i.e., similar configuration) under four-point bending exhibited a slightly lower improvement in peak capacity. For example, Beam 1P-GFRP-SB had a 15.18% improvement in its capacity, whereas the same beam tested under four-point bending (i.e., Beam 2P-GFRP-SB) had a 7.22% improvement in its capacity. Beams in Group B demonstrated relatively higher improvements in peak capacities. The greatest improvement was observed in the case of a U-shaped configuration of LC-GFRP applied along the full span and supplemented with through-bolt anchors.

Table 3 also provides the energy dissipation capacity of all beams and their improvements with respect to their corresponding control beams. The improvement in dissipated energy in Group A beams was significantly lower than in Group B beams. This is because of the following two reasons: (1) two beams in Group A were tested under three-point bending, and (2) no anchors were employed to supplement external confinement. The highest improvement in dissipated energy was observed for Beam 1P-GFRP-U, corresponding to an increase of 114%. On the contrary, an increase of up to 4562% was observed in Group B beams. This highlights the importance of anchors in resisting shear strength and improving the shear capacity of RC beams. The highest improvement imparted by LC-GFRP sheets was 2945%, corresponding to an FW configuration. In terms of the initial stiffness, it did not vary significantly and remained between 8.00 kN/mm and 9.00 kN/mm for beams tested under four-point bending.

### 3.4. Effect of Anchors on Peak Capacity

**Figure 18:**
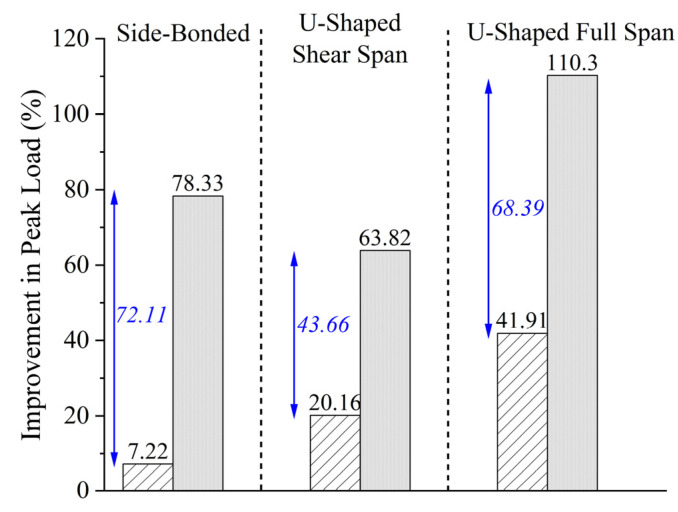
Comparison of increase in load for beams with and without through-bolt anchors.

We report that the beams in Group A were strengthened without the additional aid of through-bolt anchors. The efficacy of the through-bolt was assessed by comparing the results in both groups of beams. For the sake of similarity in loading conditions, only the results of beams under four-point bending were compared in Figure 18. The effect of anchors on the gain in peak capacity is evident for every configuration type. It is interesting to observe that the improvement in capacity due to a side-bonded configuration (with anchors) was 78.33%, whereas the same improvement from a U-shaped configuration (with anchors) was 63.82%. Moreover, the highest gain was observed in the case of a U-shaped configuration along the full span. However, this was not accompanied by an equal improvement in ductility. Hence, a side-bonded with anchors or U-shaped with anchors configuration resulted in the optimum improvement in the behavior of shear-critical beams. It must be noted that shear reinforcement in the form of stirrups was not provided in Group A beams. Therefore, that effect could have played an important role in determining the improvement in peak capacity.

### 3.5. Strain Gauge Measurements

**Figure 19:**
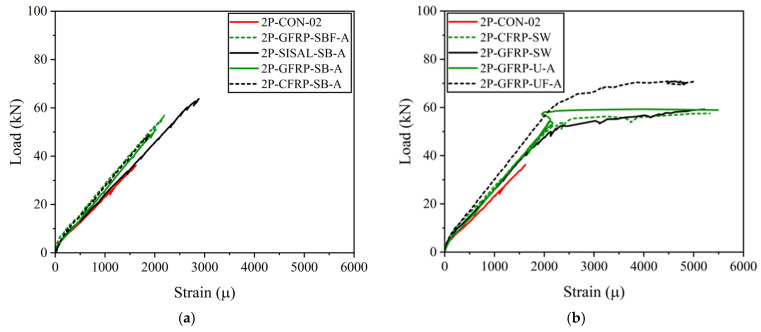
Comparison of strain of longitudinal steel bar (**a**) without yielding and (**b**) with yielding.

A single strain gauge was attached to the bottom longitudinal bar at the midspan of the beams in Group B. The recorded strain measurements are shown in Figure 19a for the beams without yielding steel bars. Beams 2P-CON-02, 2P-GFRP-SB-A, 2P-GFRP-SBF-A, 2P-CFRP-SB-A, and 2P-SISAL-SB-A did not yield at failure, and this was reflected in their load–deflection curves and failure modes. In general, side-bonded configurations did not prevent shear failure completely. Consequently, their load–deflection curves exhibited a slight improvement in ductility. As shown in Figure 19a, the maximum strain in these beams was higher than the control beam, but a yield plateau could not be observed. Figure 19b shows longitudinal strain variation for beams that did not fail in shear strength. In addition, Beam 2P-GFRP-UF-A also exhibited yielding. The maximum strain was lower than the fracture strain, ascribed to the failure of strain gauges (Figure 19b).

Natural fiber-reinforced composites, as a critical reinforcement material, warrant thorough analysis of their performance and advantages. Comparatively, CFRP, although costly, boasts superior mechanical properties, outstanding fatigue resistance, and corrosion resistance [41,42]. GFRP offers a more budget-friendly option with good mechanical characteristics but tends to become brittle over time in alkaline concrete environments. Similarly, natural fiber composites offer numerous benefits, including environmental friendliness and abundant sources, yet their performance in alkaline-abundant environments is yet to be investigated. Therefore, further studies are needed to address this issue and explore the confinement effectiveness of LC-GFRP composites in the presence of corrosion-prone conditions.

## 4. Analytical Investigations on Shear Capacity of LCGFRP-Strengthened Beams

**Figure 20:**
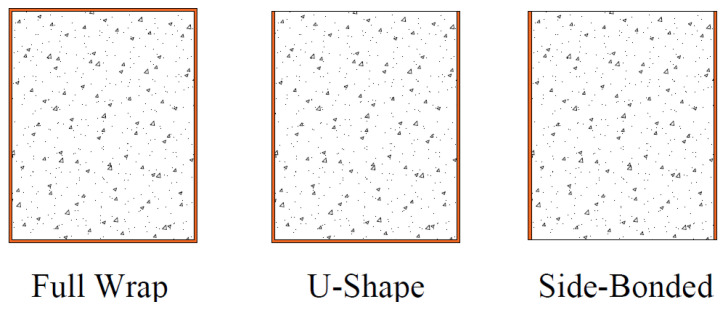
Configuration schemes for the strengthening of beams.

Figure 20 showcases three unique Fiber-Reinforced Polymer (FRP) wrapping configurations specifically developed to amplify the shear strength of prismatic rectangular beams or columns. Column applications benefiting from access on all four sides adopt a comprehensive FRP wrapping strategy. Conversely, beam applications, constrained by an integral slab that obstructs full wrapping, can enhance shear strength through the selective application of FRP wrapping or bonding on two opposing sides of the member. Experimental results showed that all techniques proved to be effective in enhancing the shear strength of beams. Among these approaches, the most efficient method was the complete wrapping of the section, followed by the three-sided U-wrap technique. On the other hand, bonding to two sides of the beam was found to be the least efficient approach in terms of shear strength improvement. Similar observations have been documented elsewhere [43]. The experimental contributions of LC-GFRP wraps to shear strength enhancement were computed by subtracting the peak capacity of strengthened beams from that of the control beam.

As per the ACI 440.2R-17 guideline [43], the shear contribution ($V_{f}$) of Fiber-Reinforced Polymer (FRP) shear reinforcement can be determined using Equation (1.1) (see Table 4). The area of the FRP external reinforcement ($A_{f}$) and the effective stress in the FRP, represented by the stress level at section failure ($f_{fe}$), are computed through Equations (1.2) and (1.4), respectively. In the case of fully covered (wrapped) elements, the effective strain level in the FRP reinforcement ($\epsilon_{fe}$) must adhere to the accepted limit of 0.004, as described in Equation (1.4). Furthermore, in situations where continuous fiber wrapping is employed, the FRP spacing ($S_{f}$) should be equal to the fiber width ($w_{f}$). As per the ACI 440 [43] guidelines, the maximum allowable design strain for CFRP laminate used in the shear strengthening of RC beams is set at 0.004. In this particular research, a similar approach has been adopted, and the maximum strain for LC-GFRP laminate has also been considered as 0.004. Furthermore, ACI 440.2R-17 [43] applies reduction coefficients on the effective strain $\epsilon_{fe}$ to account for the delamination in U-wrap or side-bonded configurations. In this study, delamination was not observed when a U-shaped configuration was adopted in conjunction with through-bolt anchors, i.e., Beam 2P-GFRP-U-A. Therefore, the present analytical work is limited to the case of U-shaped configurations with anchor bolts only. Furthermore, specimens with full wrapping, i.e., beams 2P-GFRP-SW and 2P-GFRP-FW, were also considered. 

In the Chen and Teng model [44], the FRP shear strengthening contribution to the shear strength of the RC member can be mathematically expressed, as presented in Equation (2.1) and Table 4. This model is based on fracture mechanics and considers two distinct failure modes for shear capacity evaluation: fiber rupture and fiber debonding. However, only the failure mode via the rupture of LC-GFRP is considered. To ensure strain compatibility, it is recommended that $\epsilon_{max}$ be set to 1.5% if no other specific recommendations are available. 

In accordance with the TEC-18 [45], the contribution of FRP shear reinforcement, denoted as $V_{f}$, can be calculated using Equation (3.1), as presented in Table 4. This equation comprises several parameters, such as the number of FRP winding layers on a single concrete surface ($n_{f}$), the effective thickness of the FRP layer ($t_{f}$), the width of the FRP strip ($w_{f}$), the elasticity modulus of the FRP ($E_{f}$), the effective unit elongation limit ($\epsilon_{f}$), the effective depth of section d, and the center-to-center spacing of FRP strips ($s_{f}$). Similarly to the guidelines in ACI 440, when continuous fiber wrapping is utilized, the value of FRP spacing ($s_{f}$) must be set to be equal to the value of the fiber width ($w_{f}$). It is important to highlight that within this regulation, the only accepted wrapping technique is the full wrapping technique. Additionally, the code does not account for the direction of the fiber angle, and the fiber direction is always assumed to be at 90 degrees.

In the context of the shear strengthening of RC beams, as outlined in fib-TG 9.3 [46], the shear capacity of the reinforced beam can be determined through the utilization of Equation (4.1), as presented in Table 4. When dealing with FRP fully wrapped configurations, the design value of the effective FRP strain ($\epsilon_{fd,e}$) can be calculated following the formulation presented in Equation (4.2). To ascertain the FRP reinforcement ratio ($\rho_{f}$) for continuously bonded shear reinforcement, considering the thickness ($t_{f}$), Equation (3.3) is employed. For cases where FRP reinforcement is presented in the form of strips or sheets with a particular width ($b_{w}$) at spacing ($s_{f}$), the ratio can be computed using Equation (3.4). Furthermore, it should be noted that the elastic modulus of the FRP in the principal fiber orientation ($E_{f}$) is expressed in GPa.

### Comparison of Experimental and Predicted Results

Table 5 presents the experimental and predicted shear contributions from LC-GFRP sheets. In general, the models by ACI 440.2R-17 [43] and fib-TG 9.3 [46] yielded close predictions for Beam 2P-GFRP-U-A, with predicted to experimental ratios of 1.06 and 1.04, respectively, whereas the models by Chen and Teng [44] and TEC-18 [45] slightly underestimated the shear contribution of LC-GFRP to shear strength. All of the models underestimated the shear contribution of LC-GFRP strips. This suggests that the models of ACI 440.2R-17 [43], Chen and Teng [44], TEC-18 [45], and fib-TG 9.3 [46] yielded ratios of 0.47, 0.39, 0.34, and 0.57, respectively. Furthermore, the maximum design effective strain proposed by ACI 440 [43], i.e., 0.004, can be utilized as the optimal design strain for improving the shear capacity of an RC beam by employing LC-GFRP confinement. To maintain a conservative approach, a 45-degree shear crack inclination was incorporated and subsequently validated through experimental investigations. However, these investigations were limited to the U-shaped configuration only and supplemented with through-anchor bolts. It is recognized that although existing models predicted the shear contribution of U-shaped LC-GFRP confinement supplemented by through-bolt anchorage with reasonable accuracy, there is a need to develop separate expressions for LC-GFRP confinement, especially when configurations other than a U-shape are employed. Generally, the predictions by ACI 440.2R-17 were higher compared to the predictions by other models. For instance, the predictions by ACI 440.2R-17, Chen and Teng, TEC-18, and fib-TG 9.3 for Beam 2P-GFRP-U-A were 24.46 kN, 20.14 kN, 17.78 kN, and 24.06 kN, respectively.

## 5. Discussions

The control Beam 2P-CON-02 exhibited brittle shear failure with noticeable shear cracks. The failure of beams 2P-GFRP-SB-A and 2P-GFRP-SBF-A was accompanied by debonding of LC-GFRP on one end, and noticeable shear cracks were observed, suggesting that only a partial improvement in the shear behavior was obtained. The failure of Beam 2P-GFRP-U-A did not exhibit debonding or shear cracks. This indicates that two layers of GFRP applied in a U-shaped configuration and supplemented with through-bolt anchors effectively prevented brittle shear failure. In comparison, the ductility of Beam 2P-GFRP-SBF-A was lower than Beam 2P-GFRP-SB-A. Nonetheless, both beams suffered shear failure, as can be seen in their abrupt drop in capacities. This implies that the application of side-bonded LC-GFRP was insufficient to alter the brittle failure to become a ductile failure. The performance of the side-bonded configuration, either along the full span or within the shear span only, was inferior to the U-shaped configuration, especially in terms of the ductility achieved. A U-shaped configuration along the full span imparted a significant increase in the peak load, but it increased the shear demand as well, resulting in an abrupt drop in load capacity. It may be noted that the provision of either U-shaped or side-bonded configurations outside the shear strength had adverse effects on the performance of LC-GFRP confinement. It is acknowledged that current models give reasonably accurate predictions for the shear contribution of U-shaped LC-GFRP confinement, along with through-bolt anchorage. However, there is a requirement to develop distinct expressions for LC-GFRP confinement, particularly when different configurations other than the U-shape are used.

## 6. Conclusions

An experimental framework comprising a total of seventeen beams was designed to explore the effects of loading type, configuration type, and the effect of less explored through-bolt anchorage on the performance of LC-GFRP confinement. Beams were tested under three-point and four-point bending, whereas LC-GFRP was applied in U-shaped, side-bonded, and fully wrapped configurations with and without through-bolt anchorages. The following conclusions could be drawn from the experimental results.

1. In three-point bending tests, LC-GFRP-reinforced beams in side-bonded and U-shaped configurations without anchors displayed no shear failure or debonding, whereas beams subjected to four-point bending without anchors exhibited shear failure and debonding of LC-GFRP wraps. Beams strengthened with LC-GFRP wraps and anchors showed varying results, with side-bonded configurations experiencing debonding and shear failure, and U-shaped configurations applied solely to the shear span successfully preventing shear failure, but full-span U-shaped configurations with anchors experiencing shear failure. Notably, the use of these wraps, especially on the tension side, also contributed to flexural capacity, potentially leaving the flexural-to-shear capacity ratio unchanged after strengthening and the member still vulnerable to shear issues.
2. The use of LC-GFRP and CFRP applied continuously or as strips on all four sides of the beam effectively prevented shear failure, eliminating the need for additional through-bolt anchors. Among the considered strengthening methods, complete wrapping of the section proved to be the most efficient, followed by the three-sided U-wrap technique, while bonding to only two sides of the beam was the least efficient. Regarding peak capacity enhancement, side-bonded, U-shaped, and U-shaped configurations along the full span with anchors yielded enhancements of 72.11%, 43.66%, and 68.39%, respectively, compared to their counterparts without anchors.
3. It is recognized that the performance of U-shaped wraps on an RC member is inferior to that of a fully wrapped member [43]. However, it was established that a combination of a U-shaped configuration in combination with through-bolt anchors yielded an equally satisfactory performance as that imparted by wraps on all four sides, mainly in terms of peak capacity improvement and ductility.
4. Existing models were analyzed to predict shear strength enhancements imparted by LC-GFRP for U-shaped with anchors and fully wrapped strip configurations. It is acknowledged that current models give reasonably accurate predictions for the shear contribution of U-shaped LC-GFRP confinement, along with through-bolt anchorage. However, there is a requirement to develop distinct expressions for LC-GFRP confinement, particularly when different configurations other than the U-shape are used.

## 7. Lessons Learned and Recommendations for Future Research

Beams strengthened with LC-GFRP wraps and anchors showed varying results, with side-bonded configurations experiencing debonding and shear failure, and U-shaped configurations applied solely to the shear span successfully preventing shear failure, but full-span U-shaped configurations with anchors experiencing shear failure. Among the considered strengthening methods, complete wrapping of the section proved to be the most efficient, followed by the three-sided U-wrap technique, while bonding to only two sides of the beam was the least efficient. In this research, the efficiency of LC-GFRP was observed to be higher than the CFRP and Sisal FRPs. However, there is a need to further explore the use of LC-GFRP in comparison with other FRPs for other structural members such as columns, joints, and slabs.

## Figures and Tables

**Table 1:** Properties of LC-GFRP, CFRP, Sisal, and epoxy resin.

Composite	Ultimate Strength (MPa)	Ultimate Strain (%)	Elastic Modulus (GPa)
Epoxy	17.20	0.63	2.72
LC-GFRP	377.64	2.04	18.70
CFRP	350.00	1.50	250.00
Sisal	79.43	5.65	13.79

**Table 2:** Summary of all specimens.

Group	Beam Type	Beams	Fiber	Fiber Layers	Anchor
A	Type-01	1P-CON	-	-	-
		1P-GFRP-SB	GFRP	2 Layers	No
		1P-GFRP-U	GFRP	2 Layers	No
		2P-CON-01	-	-	-
		2P-GFRP-SB	GFRP	2 Layers	No
		2P-GFRP-U	GFRP	2 Layers	No
		2P-GFRP-UF	GFRP	2 Layers	No
B	Type-02	2P-CON-02	-	-	-
		2P-GFRP-SB-A	GFRP	2 Layers	Yes
		2P-GFRP-SBF-A	GFRP	2 Layers	Yes
		2P-GFRP-U-A	GFRP	2 Layers	Yes
		2P-GFRP-UF-A	GFRP	2 Layers	Yes
		2P-GFRP-SW	GFRP	2 Layers	Yes
		2P-GFRP-FW	GFRP	2 Layers	No
		2P-CFRP-SB-A	CFRP	1 Layer	Yes
		2P-CFRP-SW	CFRP	1 Layer	No
		2P-SISAL-SB-A	Sisal	2 Layers	Yes

Note: SB stands for side-bonded within the shear span, U stands for U-shaped within the shear span, UF stands for U-shaped along the full span, SBF stands for side-bonded along the full span, SW stands for strips within the shear span, and FW stands for full wraps within the shear span.

**Table 3:** Summary of experimental results.

Beams	Shear Capacity (kN)	Increase in Capacity (%)	Failure Mode	Dissipated Energy (kN-mm)	Increase in Dissipated Energy (%)	Initial Stiffness (kN/mm)
1P-CON	39.50	-	Shear failure	1618	-	5.04
1P-GFRP-SB	45.50	15.18	Flexural failure, no debonding	3075	90	5.57
1P-GFRP-U	49.40	25.06	Flexural failure, no debonding	3460	114	5.80
2P-CON-01	42.35	-	Shear failure	1669	-	5.92
2P-GFRP-SB	47.46	7.22	Shear failure, debonding	1707	2	8.50
2P-GFRP-U	50.44	20.16	Shear failure, debonding	2009	20	8.70
2P-GFRP-UF	56.06	41.91	Shear failure, debonding	2180	31	10.74
2P-CON-02	36.20	-	Shear failure	431	-	8.93
2P-GFRP-SB-A	56.57	78.33	Debonding at one end, shear cracks	1527	254	8.60
2P-GFRP-SBF-A	56.83	56.99	Debonding at one end, shear cracks	953	121	8.52
2P-GFRP-U-A	59.30	63.82	Flexural, no debonding	2253	423	8.71
2P-GFRP-UF-A	76.16	110.30	Shear failure, debonding	1643	281	8.86
2P-GFRP-SW	63.02	74.08	Flexural, no debonding	2279	429	8.15
2P-GFRP-FW	59.64	64.76	Flexural, no debonding	2945	583	8.00
2P-CFRP-SB-A	49.19	35.88	Shear failure	908	111	8.57
2P-CFRP-SW	62.15	71.68	Flexural, no debonding, concrete crushing	4562	959	8.06
2P-SISAL-SB-A	64.56	56.27	Shear failure, no debonding	1506	249	9.06

**Table 4:** Existing models to determine shear strength contribution of external FRP wraps.

Reference	General Expression	Full Wrapping Case	Comments
ACI 440.2R-17 [43]	$\begin{array}{c} V_{f}=\frac{A_{f}f_{fe}\left(\sin\left(\alpha \right)+\cos\left(\alpha \right)\right)d_{f}}{s_{f}}\;\;\left(1.1\right) \end{array}$	$\begin{array}{cc} A_{f}=2n_{f}t_{f}w_{f} & \;\;\left(1.2\right)\\ f_{fe}=\epsilon_{fe}E_{f} & \;\;\left(1.3\right)\\ \epsilon_{fe}=0.004\leq 0.75f_{u} & \;\;\left(1.4\right) \end{array}$	Only the case of full wrapping is considered, as no delamination was observed in U-shaped configuration with anchors. $s_{f}=w_{f}$ for continuous wrapping.
Chen and Teng [44]	$\begin{array}{c} V_{f}=2f_{f,eu}t_{f}w_{f}\frac{h_{frp,e}\left(\cot\left(\theta \right)+\cot\left(\alpha \right)\right)\sin\left(\alpha \right)}{s_{f}}\;\;\left(2.1\right) \end{array}$	$\begin{array}{cc} f_{fu,e}=D_{frp}\sigma_{frp,max} & \;\;\left(2.2\right)\\ h_{frp,e}=0.9d-\left(h-d_{frp}\right) & \;\;\left(2.3\right)\\ \sigma_{frp,max}=\min\left\{\begin{array}{c} 0.8f_{frp}\\ 0.8\epsilon_{max}E_{f}\;\;\;\; \end{array}\begin{array}{c} \frac{f_{frp}}{E_{f}}\leq \epsilon_{max}\\ \frac{f_{frp}}{E_{f}}>\epsilon_{max} \end{array}\right\} & \;\;\left(2.4\right)\\ D_{frp}=\frac{1+\zeta }{2} & \;\;\left(2.5\right) \end{array}\;$	Fracture mechanics-based model.
TEC-18 [45]	$\begin{array}{c} V_{f}=\frac{2n_{f}t_{f}w_{f}E_{f}\epsilon_{fe}d}{s_{f}}\;\;\left(3.1\right) \end{array}$	$\begin{array}{c} min\left\{\begin{array}{c} \epsilon_{fe}\leq 0.004\\ \epsilon_{fe}\leq 0.5\epsilon_{fu} \end{array}\right\}\;\;\left(3.2\right) \end{array}$	Does not account for side-bonded configuration.
FIB-TG 9.3 [46]	$\begin{array}{c} V_{f}=0.9\epsilon_{fd,e}E_{f}\rho_{f}b_{w}d\left(\cot\left(\theta \right)+\cot\left(\alpha \right)\right)\sin\left(\alpha \right)\;\;\left(4.1\right) \end{array}$	$\begin{array}{cc} \epsilon_{fd,e}=0.17{\left(\frac{{f_{c}^{\prime }}^{\frac{2}{3}}}{E_{f}\rho_{f}}\right)}^{0.30}\times \epsilon_{fu} & \;\;\left(4.2\right)\\ \rho_{f}=\left(\frac{2t_{f}}{b_{w}}\right)\left(\frac{w_{f}}{s_{f}}\right) & \;\;(4.3) \end{array}$	$s_{f}=w_{f}$ for continuous wrapping. $E_{f}$ in GPa.

Note: $n_{f}$ is the number of wraps, $t_{f}$ is the thickness of wraps, $A_{f}$ is the area of wraps that passes a shear crack, $s_{f}$ is the center-to-center spacing of strips, $d_{f}$ is the effective depth of wraps, $w_{f}$ is the width of strips, $f_{c}^{\prime }$ is the compressive strength of concrete, $E_{f}$ is the elastic modulus of wraps, $\epsilon_{fu}$ is the rupture strain of wraps, and $\epsilon_{fe}$ is the effective strain of wraps.

**Table 5:** Comparison of experimental and predicted shear contributions of LC-GFRP confinement.

Beam	$V_{f,exp}$ (kN)	ACI 440.2R-17			Chen and Teng		TEC-18			fib-TG 9.3		
		$V_{f,pred}$ (kN)	R	$\epsilon_{fe}$	$V_{f,pred}$ (kN)	R	$V_{f,pred}$ (kN)	R	$\epsilon_{fe}$	$V_{f,pred}$ (kN)	R	$\epsilon_{fd,e}$
2P-GFRP-U-A	23.10	24.46	1.06	0.004	20.14	0.87	-	-	0.004	24.06	1.04	0.009
2P-GFRP-SW	25.94	12.23	0.47		10.09	0.39	8.89	0.34		14.81	0.57	

## Data Availability

Not applicable.

## References

[1] Ejaz A., Ruangrassamee A., Kruavit P., Udomworarat P., Wijeyewickrema A.C. (2022). Strengthening of Substandard Lap Splices Using Hollow Steel Section (HSS) Collars. Structures.

[2] AL-Shalif S.A.H., Akın A., Aksoylu C., Hakan Arslan M. (2022). Strengthening of Shear-Critical Reinforced Concrete T-Beams with Anchored and Non-Anchored GFRP Fabrics Applications. Structures.

[3] (2019). Building Code Requirements for Structural Concrete and Commentary.

[4] Özkılıç Y.O., Gemi L., Madenci E., Aksoylu C. (2023). Effects of Stirrup Spacing on Shear Performance of Hybrid Composite Beams Produced by Pultruded GFRP Profile Infilled with Reinforced Concrete. Arch. Civ. Mech. Eng..

[5] Karimizadeh H., Arabzadeh A., Eftekhar M.R., Amani Dashlejeh A. (2022). Shear Strengthening of RC Deep Beams with Symmetrically or Asymmetrically Positioned Square Openings Using CFRP Composites and Steel Protective Frames. Adv. Civ. Eng..

[6] Mosallam A.S. (2002). Composites in Construction. Handbook of Materials Selection.

[7] Chen J.F., Teng J.G. (2003). Shear Capacity of FRP-Strengthened RC Beams: FRP Debonding. Constr. Build. Mater..

[8] Al-Sulaimani G.J., Sharif A., Basunbul I.A., Baluch M.H., Ghaleb B.N. (1994). Shear Repair for Reinforced Concrete by Fiberglass Plate Bonding. ACI Struct. J..

[9] Khalifa A., Nanni A. (2000). Improving Shear Capacity of Existing RC T-Section Beams Using CFRP Composites. Cem. Concr. Compos..

[10] Chaallal O., Nollet M.J., Perraton D. (1998). Strengthening of Reinforced Concrete Beams with Externally Bonded Fiber-Reinforced-Plastic Plates: Design Guidelines for Shear and Flexure. Can. J. Civ. Eng..

[11] Joyklad P., Waqas H.A., Hafeez A., Ali N., Ejaz A., Hussain Q., Khan K., Sangthongtong A., Saingam P. (2023). Experimental Investigations of Cement Clay Interlocking Brick Masonry Structures Strengthened with CFRP and Cement-Sand Mortar. Infrastructures.

[12] Teng J., Chen J., Smith S., Lam L. (2002). FRP: Strengthened RC Structures.

[13] Chen J.F., Teng J.G. (2001). Anchorage Strength Models for FRP and Steel Plates Bonded to Concrete. J. Struct. Eng..

[14] Chen G.M., Zhang Z., Li Y.L., Li X.Q., Zhou C.Y. (2016). T-Section RC Beams Shear-Strengthened with Anchored CFRP U-Strips. Compos. Struct..

[15] Chaiyasarn K., Poovarodom N., Ejaz A., Ng A.W., Hussain Q., Saingam P., Mohamad H., Joyklad P. (2023). Influence of natural fiber rope wrapping techniques on the compressive response of recycled aggregate concrete circular columns. Results Eng..

[16] Concrete Society (2012). TR55 Design Guidance for Strengthening Concrete Structures Using Fibre Composite Materials: Report of a Concrete Society Working Party.

[17] Teng J.G., Smith S.T., Yao J., Chen J.F. (2003). Intermediate Crack-Induced Debonding in RC Beams and Slabs. Constr. Build. Mater..

[18] Matthys S. (2000). Structural Behaviour and Design of Concrete Members Strengthened with Externally Bonded FRP Reinforcement. Ph.D. Thesis.

[19] Fanning P.J., Kelly O. (2001). Ultimate Response of RC Beams Strengthened with CFRP Plates. J. Compos. Constr..

[20] Ceroni F., Pecce M. (2009). Design Provisions for Crack Spacing and Width in RC Elements Externally Bonded with FRP. Compos. B Eng..

[21] Suparp S., Ejaz A., Khan K., Hussain Q., Joyklad P., Saingam P. (2023). Load-Bearing Performance of Non-Prismatic RC Beams Wrapped with Carbon FRP Composites. Sensors.

[22] Almasabha G., Murad Y., Alghossoon A., Saleh E., Tarawneh A. (2023). Sustainability of Using Steel Fibers in Reinforced Concrete Deep Beams without Stirrups. Sustainability.

[23] Islam M.R., Mansur M.A., Maalej M. (2005). Shear Strengthening of RC Deep Beams Using Externally Bonded FRP Systems. Cem. Concr. Compos..

[24] Triantifillou T.C. (1998). Shear Strengthening of Reinforced Concrete Beams Using Epoxy-Bonded FRP Composites. ACI Struct. J..

[25] Monti G., Liotta M. (2007). Tests and Design Equations for FRP-Strengthening in Shear. Constr. Build. Mater..

[26] Baggio D., Soudki K., Noël M. (2014). Strengthening of Shear Critical RC Beams with Various FRP Systems. Constr. Build. Mater..

[27] Boyd A.J. (2000). Rehabilitation of Reinforced Concrete Beams with Sprayed Glass Fiber Reinforced Polymers. Ph.D. Thesis.

[28] Arslan M.H., Yazman Ş., Hamad A.A., Aksoylu C., Özkılıç Y.O., Gemi L. (2022). Shear Strengthening of Reinforced Concrete T-Beams with Anchored and Non-Anchored CFRP Fabrics. Structures.

[29] Aksoylu C., Yazman Ş., Özkılıç Y.O., Gemi L., Arslan M.H. (2020). Experimental Analysis of Reinforced Concrete Shear Deficient Beams with Circular Web Openings Strengthened by CFRP Composite. Compos. Struct..

[30] Soleimani S.M., Banthia N. (2012). Shear Strengthening of RC Beams Using Sprayed Glass Fiber Reinforced Polymer. Adv. Civ. Eng..

[31] Hussain Q., Pimanmas A. (2015). Shear Strengthening of RC Deep Beams with Sprayed Fibre-Reinforced Polymer Composites (SFRP) and Anchoring Systems: Part 1. Experimental Study. Eur. J. Environ. Civ. Eng..

[32] Hussain Q., Pimanmas A. (2015). Shear Strengthening of RC Deep Beams with Openings Using Sprayed Glass Fiber Reinforced Polymer Composites (SGFRP): Part 1. Experimental Study. KSCE J. Civ. Eng..

[33] Yoddumrong P., Rodsin K., Katawaethwarag S. (2020). Seismic Strengthening of Low-Strength RC Concrete Columns Using Low-Cost Glass Fiber Reinforced Polymers (GFRPs). Case Stud. Constr. Mater..

[34] Rodsin K., Hussain Q., Suparp S., Nawaz A. (2020). Compressive Behavior of Extremely Low Strength Concrete Confined with Low-Cost Glass FRP Composites. Case Stud. Constr. Mater..

[35] Rodsin K., Ali N., Joyklad P., Chaiyasarn K., Al Zand A.W., Hussain Q. (2022). Improving Stress-Strain Behavior of Waste Aggregate Concrete Using Affordable Glass Fiber Reinforced Polymer (GFRP) Composites. Sustainability.

[36] Joyklad P., Saingam P., Ali N., Ejaz A., Hussain Q., Khan K., Chaiyasarn K. (2022). Low-Cost Fiber Chopped Strand Mat Composites for Compressive Stress and Strain Enhancement of Concrete Made with Brick Waste Aggregates. Polymers.

[37] Haroon M., Moon J.S., Kim C. (2021). Performance of Reinforced Concrete Beams Strengthened with Carbon Fiber Reinforced Polymer Strips. Materials.

[38] Kim Y., Quinn K., Satrom N., Garcia J. (2012). Shear Strengthening of Reinforced and Prestressed Concrete Beams Using Carbon Fiber Reinforced Polymer (CFRP) Sheets and Anchors.

[39] (2021). Standard Specification for Epoxy Molding Compounds.

[40] Lima P., Santos R., Ferreira S. (2014). Characterization and Treatment of Sisal Fiber Residues for Cement-Based Composite Application. Eng. Agrícola.

[41] Xian G., Guo R., Li C. (2022). Combined effects of sustained bending loading, water immersion and fiber hybrid mode on the mechanical properties of carbon/glass fiber reinforced polymer composite. Compos. Struct..

[42] Wu J., Zhu Y., Li C. (2023). Experimental Investigation of Fatigue Capacity of Bending-Anchored CFRP Cables. Polymers.

[43] (2017). Guide for the Design and Construction of Externally Bonded FRP Systems for Strengthening Concrete Structures.

[44] Chen J.F., Teng J.G. (2003). Shear Capacity of Fiber-Reinforced Polymer-Strengthened Reinforced Concrete Beams: Fiber Reinforced Polymer Rupture. J. Struct. Eng..

[45] (2018). Turkey: New Building Code for Earthquake Resilience.

[46] CEP-FIB (2001). Fib TG 9.3 Externally Bonded FRP Reinforcement for RC Structures.

